# Cardiovascular magnetic resonance of myocardial edema using a short inversion time inversion recovery (STIR) black-blood technique: Diagnostic accuracy of visual and semi-quantitative assessment

**DOI:** 10.1186/1532-429X-14-22

**Published:** 2012-03-28

**Authors:** Darach O h-Ici, John P Ridgway, Titus Kuehne, Felix Berger, Sven Plein, Mohan Sivananthan, Daniel R Messroghli

**Affiliations:** 1 Department of Congenital Heart Disease and Pediatric Cardiology, Deutsches Herzzentrum Berlin, Augustenburger Platz 1, Berlin 13353, Germany; 2 Medical Physics, Leeds General Infirmary, Great George Street, Leeds LS1 3EX, UK; 3 Leeds Institute of Genetics, Health and Therapeutics & Cardiovascular Research Centre, University of Leeds, Great George Street, Leeds LS1 3EX, UK; 4 Department of Cardiology, Leeds General Infirmary, Great George Street, Leeds LS1 3EX, UK

## Abstract

**Background:**

The short inversion time inversion recovery (STIR) black-blood technique has been used to visualize myocardial edema, and thus to differentiate acute from chronic myocardial lesions. However, some cardiovascular magnetic resonance (CMR) groups have reported variable image quality, and hence the diagnostic value of STIR in routine clinical practice has been put into question. The aim of our study was to analyze image quality and diagnostic performance of STIR using a set of pulse sequence parameters dedicated to edema detection, and to discuss possible factors that influence image quality. We hypothesized that STIR imaging is an accurate and robust way of detecting myocardial edema in non-selected patients with acute myocardial infarction.

**Methods:**

Forty-six consecutive patients with acute myocardial infarction underwent CMR (day 4.5, +/- 1.6) including STIR for the assessment of myocardial edema and late gadolinium enhancement (LGE) for quantification of myocardial necrosis. Thirty of these patients underwent a follow-up CMR at approximately six months (195 +/- 39 days). Both STIR and LGE images were evaluated separately on a segmental basis for image quality as well as for presence and extent of myocardial hyper-intensity, with both visual and semi-quantitative (threshold-based) analysis. LGE was used as a reference standard for localization and extent of myocardial necrosis (acute) or scar (chronic).

**Results:**

Image quality of STIR images was rated as diagnostic in 99.5% of cases. At the acute stage, the sensitivity and specificity of STIR to detect infarcted segments on visual assessment was 95% and 78% respectively, and on semi-quantitative assessment was 99% and 83%, respectively. STIR differentiated acutely from chronically infarcted segments with a sensitivity of 95% by both methods and with a specificity of 99% by visual assessment and 97% by semi-quantitative assessment. The extent of hyper-intense areas on acute STIR images was 85% larger than those on LGE images, with a larger myocardial salvage index in reperfused than in non-reperfused infarcts (p = 0.035).

**Conclusions:**

STIR with appropriate pulse sequence settings is accurate in detecting acute myocardial infarction (MI) and distinguishing acute from chronic MI with both visual and semi-quantitative analysis. Due to its unique technical characteristics, STIR should be regarded as an edema-weighted rather than a purely T2-weighted technique.

## Background

The ability of magnetic resonance (MR) techniques to assess myocardial infarction has attracted the interest of cardiovascular researchers from the very beginning of cardiovascular MR (CMR). The behavior of acute myocardial infarction (MI) in dogs was described by Higgins as early as 1983 and both T1 and T2 were found to increase after coronary occlusion [1]. Wisenberg et al. observed that this increase starts in the first hour after myocardial reperfusion [2]. In 1988, Been et al. published T1 maps of patients with acute myocardial infarction demonstrating elevated T1 values in areas of acute MI [3]. It could be shown that these increases of relaxation times are due to myocardial edema [1,4], which is regarded as a phenomenon that is invariably linked to acute myocardial injury.

A new approach for the visualization of myocardial edema in humans was introduced in 1996. In their work, Simonetti adapted the short time-from-inversion inversion recovery (STIR) black-blood technique for cardiac applications [5] and showed examples of clinical patients, where the areas of acute infarction could be clearly visualized with this technique as regions with hyper-intense signal. Only a limited number of CMR centers used this approach, until Abdel-Aty published in 2004 the results of a study comparing STIR images in acute and chronic myocardial infarction [6]. In this study, STIR images had a specificity of 96% to differentiate patients with acute from those with chronic MI. The authors concluded that an imaging approach combining late gadolinium enhancement (LGE) and T2-weighted CMR accurately differentiates acute from chronic MI.

Since STIR was perceived as a T2-weighted technique, several other publications subsequently investigated alternative T2 techniques to assess myocardial edema. Some authors speculated that the hyper-intense area on T2-weighted techniques represents the area at risk, and several animal studies support this concept [7,8].

Edema imaging has been shown to be also useful in other acute myocardial diseases, such as transplant rejection [9], myocarditis [10], and stress-induced (Takotsubo) cardiomyopathy [11].

The centers involved in these studies have translated the results from their research into their clinical practice, whereas some other centers aiming to do the same have experienced major discrepancies between their findings with T2-weighted techniques and clinical status of their patients. Specifically, a large prevalence of image artifacts and a significant variation in results depending on the choice of pulse sequence parameters has been reported [12], leading to a considerable amount of uncertainty and confusion among CMR centers and physicians new to edema imaging.

With this discussion in mind, we set to investigate if the practice to use STIR for the detection of myocardial edema in both clinical and research settings was justified. The aim of this study was therefore to review the technical principles behind STIR, and to assess the diagnostic accuracy of STIR in patients who underwent CMR in both the acute and chronic stages after MI. We hypothesized that STIR imaging is an accurate and robust way of detecting myocardial edema in non-selected patients with acute myocardial infarction.

## Methods

Over a period of 14 months, 46 consecutive patients with acute first MI were prospectively enrolled to undergo CMR within the first week and at 6 months after MI at the Leeds General Infirmary, Leeds, UK. Exclusion criteria included clinical instability and the normal contraindications to CMR or the administration of gadolinium (Gd) contrast; no other exclusion criteria were applied. The local ethics committee approved the study, and written informed consent was obtained from all patients.

### MRI protocol

CMR studies were performed on a 1.5 Tesla clinical MR system (Gyroscan Intera CV; Philips Healthcare, Best, The Netherlands) with a 5-element cardiac phased-array coil. The imaging protocol included cine, STIR, and LGE imaging at standardized apical, mid-cavity, and basal short-axis levels [13] covering 16 segments of the 17-segment model as recommended by AHA [14].

All images were acquired with identical geometric parameters (acquired pixel size 1.8 × 2.3 mm, reconstructed pixel slice 1.5 × 1.5 mm, slice thickness 8 mm) during end-expiration breathhold period. For cine imaging, a balanced steady-state free precession (SSFP) pulse sequence was used (TR 3.5 ms, TE 1.7 ms, FA 55°, 18 phases). For STIR, we used a triple-IR black-blood turbo-spin echo pulse sequence with inversion time (TI) 180 ms, TSE factor 32, bandwidth 504 Hz, half scan factor 0.65, TE 100 ms, TR 2 RR intervals for heart rates ≤ 80/min or 4 RR for heart rates > 80/min, coil sensitivity-based homogeneity correction (CLEAR), and 2 signal averages. Twelve minutes after intravenous application of a standard MRI contrast agent (gadopentetate dimeglumine - Magnevist, Schering AG, Berlin, Germany; 0.15 mmol/kg), 4 test scans were performed with increasing TI, before definitive LGE images were acquired with an IR-prepared gradient echo technique (TR 4.7 ms, TE 2.0 ms, FA 15°, 2 signal averages) using the TI that allowed for the best nulling of normal myocardium.

### Image analysis

**Figure 1:**
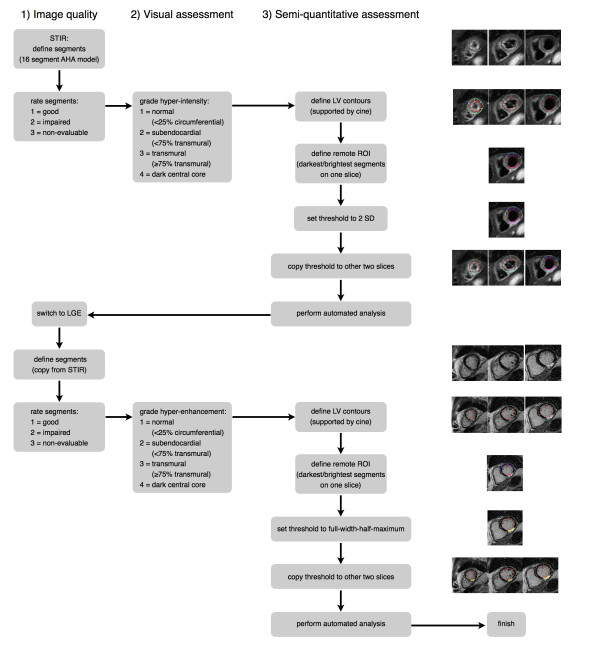
**Flow chart for analysis of STIR and LGE images**.

Offline image analysis was performed with dedicated software (CMR 42, Circle Cardiovascular Imaging, Calgary) by one observer (D.O.H, cardiologist, 2 years of CMR experience) blinded to infarct age and location. Figure 1 illustrates the different steps of the analysis. For each of the 16 myocardial segments, image quality (good, no artifacts = 1, impaired, minor artifacts = 2, non-evaluable, major artifacts = 3) and presence of hyper-intensity (none = 1, subendocardial = 2, transmural = 3, dark central core = 4) were assessed visually for both STIR and LGE images. On these images, subepicardial and subendocardial contours of the left ventricle (LV) myocardium were manually traced. In case of ambiguity regarding these contours, cine images of corresponding slice positions were assessed to differentiate LV myocardium from the intense signal caused by slow-flowing blood. To perform semi-quantitative assessment, intensity thresholds were set (2 standard deviations (SD) for STIR [15], full width at half maximum for LGE [16,17]) based on the signal intensity of normal-appearing myocardium. Areas with signal intensities above these thresholds were automatically delineated. Central dark areas (hypo-enhanced cores) within the areas of high signal intensities were manually included into the high-signal areas. Total area at risk was expressed as percentage of the LV volume, given by the sum of the volume of high signal intensity on STIR (edema) for all slices divided by the sum of the LV myocardial cross-sectional volumes (%LV). Correspondingly, total infarct area was expressed as percentage of the LV volume, given by the sum of the volume of high signal intensity on LGE (necrosis/scar) for all slices divided by the sum of the LV myocardial cross-sectional volumes (%LV). Myocardial salvage index (MSI) was determined as the difference between the area at risk and the total infarct area divided by the area at risk [18].

All images were analyzed on a segmental and per-patient basis. If segments were considered uninterpretable, they were excluded from the analysis. LGE data of a subset of the patients have been used in previous publications [19,20]. Infarct localization was derived from the ECG. The remote region was selected as a segment without hyperintensity or LGE manually for the visual assessment, and automatically by the software program for the semi-quantitative assessment. This was typically in a segment furthest from the hyperintense/LGE segments.

### Statistical analysis

Continuous variables are expressed as mean ± standard deviation. Results from STIR and LGE analysis were compared using Student's t test. A p-value < 0.05 was considered significant. All analysis was performed using STATA version 10 (StataCorp, College Station/TX, USA).

## Results

### Clinical data

Forty-six consecutive patients (83% male, age 57 ± 12 years) were prospectively studied in the acute stage (4 ± 1 days). Thirty (65%) of these were available for follow-up CMR at 6 months (196 ± 39 days). On ECG, localization of infarct was anterior in 44%, inferior in 54%, and posterolateral in 2%. Most cases (93%) presented with ST elevation. Creatine kinase (CK) levels were 1875 ± 1966 U/l (range 344 to 11719 U/l). 63% of patients had received revascularization therapy, which was successful according to clinical and ECG criteria in 47% of cases.

### Image quality

STIR Image quality was good in 97.8% of segments (720/736) and was uninterpretable in only four segments (0.5%) in the acute stage, and good in 98.3% (472/480) and uninterpretable in four segments (0.8%) at 6 months. The uninterpretable segments were excluded on the basis of artifact. LGE image quality was good in 98.5% of segments (725/736) and interpretable in all segments at the acute stage. At 6 months LGE image quality was good in 95.8% of segments (460/480) and interpretable in all segments.

### Image analysis

**Figure 2:**
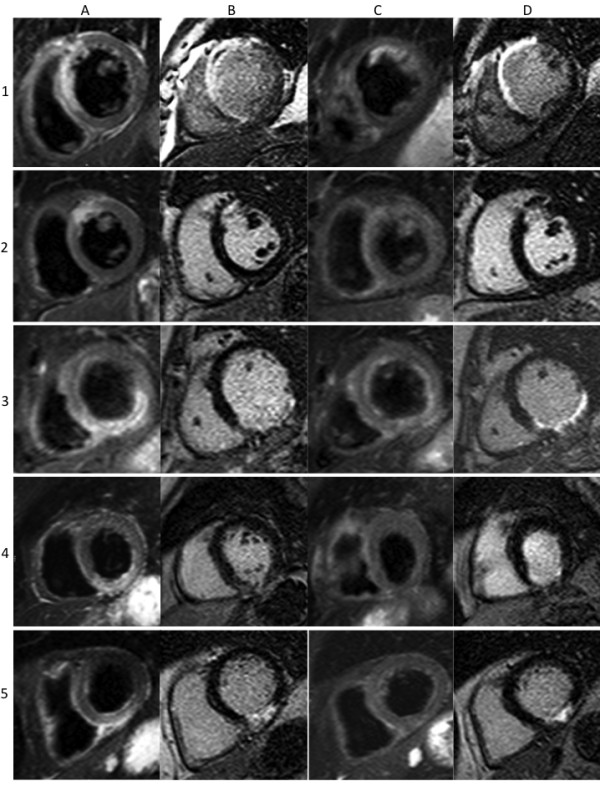
**Matched diastolic STIR and LGE images obtained in 5 patients post MI (A = acute STIR, B = acute LGE, C = 6-month STIR, D = 6-month LGE)**. Transmural edema can be seen in all acute STIR images, which disappears by 6-months. Hyperintense areas with central hypointensity on acute STIR imaging are seen in the first and third patients. These hypointense areas correspond with areas of microvascular obstruction seen on the corresponding LGE images and are thought to represent myocardial hemorrhage.

Hyperintense myocardial segments were present in all subjects on STIR images acutely and corresponded to the vascular territory downstream of the occluded coronary artery at angiography (Figure 2). At the acute stage, the per-segment sensitivity and specificity of STIR on visual assessment to detect infarcted segments was 95% and 78% respectively, and on semi-quantitative assessment was 99% and 83% respectively.

**Figure 3:**
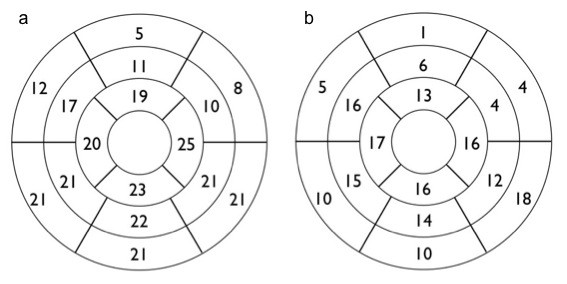
**(a) Bullseye of AHA 17-segment model demonstrating the segments with STIR hyperintensity**. (b) Bullseye of AHA 17-segment model demonstrating the segments with LGE hyperenhancement.

The distribution of the segments with hyperintensity on STIR imaging and LGE can be seen in Figure 3. It can be clearly seen that the segments affected are not focused in any territory.

Tables 1 and 2 give the segmental extent of hyperintensity on STIR and LGE for the acute and 6-months studies, respectively. The extent of hyperintense areas on acute STIR images was 84% larger than that of LGE (28.3 ± 14.1 vs. 15.4 ± 11.3%, p < 0.0001). MSI was 42% larger in reperfused than in non-reperfused infarcts (0.61 ± 0.26 vs. 0.43 ± 0.24, p = 0.035).

**Table 1:** Number of segments (%) with different degrees of hyperintensity (STIR) or LGE on visual and semi-quantitative assessments at the acute stage

	STIR visual	STIR semi-quantitative	LGE visual	LGE semi-quantitative
none	438 (60)	413 (56)	561 (76)	555 (75)
subendocardial	51 (7)	74 (10)	38 (5)	49 (7)
transmural	208 (28)	217 (30)	87 (12)	89 (12)
transmural with dark core	35 (5)	28 (4)	50 (7)	43 (6)

**Table 2:** Number of segments (%) with different degrees of hyperintensity (STIR) or LGE on visual and semi-quantitative assessments at the 6-month stage

	STIR visual	STIR semi-quantitative	LGE visual	LGE semi-quantitative
none	468 (98.3)	468 (98.3)	347 (72)	355 (74)
subendocardial	1 (0.2)	1 (0.2)	33 (7)	37 (8)
transmural	7 (1.5)	7 (1.5)	100 (21)	85 (18)
transmural with dark core	0	0	0	0

**Figure 4:**
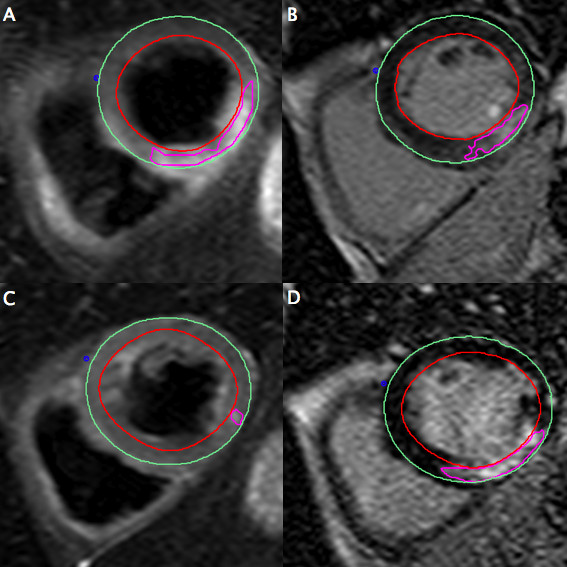
**Example of persistent hyperintense signal on STIR at 6-month follow-up**. Matched diastolic images obtained in a 76-year-old male with reperfused inferior MI. The acute images demonstrate a large area at risk on STIR (A) with a smaller infarcted zone (B). The images at 6 months show the infarct zone has has become slightly larger (D), whereas the STIR images (C) show near homogenous signal distribution but a small remaining area of hyperintensity.

On a segmental basis, STIR differentiated acute from chronic infarcts with a sensitivity of 95% by both methods and a specificity of 99% by visual assessment and 97% by semi-quantitative assessment. On the STIR images at six months, by visual assessment, three patients had STIR segments exhibiting hyper-intensity; by semi-quantitative assessment, four patients had STIR positive segments. In the three patients with persistent hyper-intensity on visual assessment, the hyperintense segments correlated with infarcted segments, and had been hyperintense at the acute stage. Potential reasons for persistent myocardial oedema could include increased wall stress and/or residual/recurring ischemia, alterations in drainage of the infarcted segments as a result of vessel damage or continuing tissue repair. In all cases the number of hyperintense segments was less than at the acute stage (Figure 4). In the one remaining patient, semi-quantitative assessment identified the septum as having a higher signal, a false positive finding not seen on visual assessment.

In order to compare samples from data that were not interdependent, cases were split into a chronic group (those cases where a follow-up CMR was available) and an acute group (all other cases). Comparing 16 acute and 30 chronic infarcts from 46 patients, acute MI was identified with a sensitivity of 100% for both methods and a specificity of 90% and 87% for visual assessment and semi-quantitative assessment, respectively.

Looking at all cases, 50 segments (7%) with hypoenhanced cores were seen on LGE images. On STIR imaging, 35 segments (5%) had hypointense cores. These segments matched in 91% of STIR images (31 cases). In the other cases, there were 4 segments on STIR imaging without matching LGE hypoenhancement, and 19 segments with hypoenhancement on LGE without corresponding STIR hypointensity. Hypoenhanced or hypointense cores were only found in acute cases.

## Discussion

Our study shows that STIR imaging with appropriate pulse sequence parameters allows for visualization and semi-quantitative assessment of myocardial edema due to acute myocardial infarction with high diagnostic accuracy.

The results of our study demonstrate the high image quality that can be achieved if a STIR sequence with appropriate pulse sequence properties is available. In our patients, we could not only assess transmurality of myocardial edema, but also clearly visualize hypointense cores within the edematous zone, a phenomenon that is related to hypoenhanced cores on LGE images [21,22], probably represents intramyocardial hemorrhage [23] and recently has been shown to be linked to adverse clinical outcome [24]. Image quality was also sufficient to assess the presence of edema on the segmental level rather than case-by-case. As a limitation, it has to be kept in mind that most patients in our study suffered from ST-elevation MI and therefore presented with severe myocardial lesions. Results might differ in patients with less severe ischemic or non-ischemic events.

This study confirms the high accuracy of STIR for differentiating acute from chronic cases of myocardial infarction that was first reported in the study by Abdel-Aty et al. [6] In contrast to that study we found that hyperintensity on STIR was not always transmural, indicating superior spatial resolution of our image data. As MSI was larger in reperfused than in non-reperfused infarcts, the hyper-intense areas in our STIR images can be attributed to the area at risk. The finding that the area at risk does not necessarily have fully transmural extent is supported by the observation that not all non-reperfused infarcts lead to transmural necrosis on LGE, and might be explained by the development of significant collateral blood supply stimulated by repeated transitory ischemia in some individuals prior to the final occlusion of an epicardial coronary vessel.

The increase in T2 associated with edema in acute MI is relatively small (25), and estimated to be on the order of 25-50% for T2 in range 45-50 ms for normal myocardium and 60-65 ms for acute MI [25]. Thus signal uniformity is critical for determination of the size of elevated T2 region. In this case we were able to benefit from the coil sensitivity-based homogeneity correction that comes with CLEAR.

**Figure 5:**
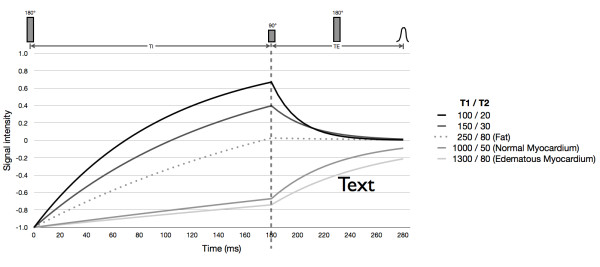
**(according to Dwyer et al., Radiology 1988; adjusted to application in myocardial imaging at 1.5 T with TI 180 ms, TE 100 ms, assuming long TR allowing full relaxation)**. STIR pulse sequence scheme (top) and its effect on net magnetization vectors for 5 categories of tissues with increasing T1 and T2. The first part (left of the vertical dashed line) shows the recovery of the longitudinal components (M_z_) in the interval between the initial STIR 180° and the TSE 90° pulses, the second part (right of the dotted line) shows the decay of the transversal components (M_xy_) after the 90° pulse. M_z _of tissues A and B with short T1 times (T1/T2 100/20 and 150/30 ms) reach positive values during the TI interval, with the shorter T1 yielding higher M_z _(T1 weighting). Following the 90° pulse, the difference (= contrast) between signal intensities is diminished during the long TE interval (T2 weighting). The signal of tissue C (250/80 ms, e.g. fat) is cancelled due to zero-crossing at the time of the 90° pulse. M_z _of tissues D and E with long T1 (1000/50 and 1300/80 ms; e.g. remote myocardium and edematous myocardium at 1.5 T, respectively [20,33]) are still negative by the time of the 90° pulse. The following T2 decay further adds to their contrast (additive effect of STIR in edema imaging).

The STIR technique is based on a double-inversion recovery black-blood turbo spin echo (TSE; equivalent: fast spin echo) pulse sequence, into which a third 180° inversion pulse is integrated. The specific feature of this third inversion pulse is its relatively short TI (140-180 ms), which leads to a suppression of the signal from fatty tissue and from anything else with a T1 relaxation time of approximately 200-250 ms. As illustrated by Dwyer et al in 1988 [26], the second effect of this inversion pulse is far more important in edema imaging: the early inversion of the relaxation curve before the TSE 90° pulse leads to an inversion of the effects of long T1 on signal intensity. The presence of increased tissue water with edema causes an increase in both T1 and T2 relaxation times. In comparison to a standard TSE sequence (without an inversion pulse), where an increase in T1 value causes a reduction in signal intensity, the inversion of the net magnetization prior to the TSE 90° pulse in STIR sequences results in an increased signal intensity for longer T1 relaxation values, provided the relaxation curves do not cross zero. If T2 weighting is also introduced by increasing the echo time, TE, the relative signal intensity is increased for increased T2 relaxation values. So in the case of a standard TSE pulse sequence, the contrast arising from T1-weighting opposes the contrast arising from T2 weighting, while in the case of a T2-weighted STIR pulse sequence, the T1 and T2 effects of water are combined which boosts the contrast between edematous and non-edematous tissue beyond the limits of pure T2 weighting (Figure 5). The above signal dependencies translate to image pixel intensities provided that a magnitude (or modulus) image display is chosen that ignores the sign of the magnetization prior to readout by the 90° pulse.

While these theoretical considerations clearly show that the STIR pulse sequence is favorable for the visualization of myocardial edema, at the moment the theoretical advantages are not realized in all CMR centers. A possible reason for this might be the lack of standardization of STIR pulse sequence parameters. From our experience there are numerous pulse sequence parameters in STIR that need to be set appropriately in order to optimize image quality and maximize the edema contrast. The list of these parameters includes, among others, timing and design of STIR and black-blood inversion pulses; quality of TSE read-out pulses; bandwidth; and slice thickness. Dwyer et al. also addressed the importance of allowing a sufficient repetition time (TR), when they systematically investigated signal evolution in STIR and predicted peak T1 values for additive T1 and T2 behavior at a given TR [26]. In practice, TR for myocardial imaging at 1.5 Tesla should be set to two RR intervals for heart rates ≤ 80/min, and to > 2 RR intervals for heart rates above that value. As most TSE implementations do not allow for such values, the same effect can be achieved by prescribing half of the given heart rate, which will lead the MR system to omit every other heart beat and thus effectively double the TR, for example, if the patient's heart rate is 90, prescribing a scanner heart rate of 45 would double the TR. While the choice of a long (> 80 ms) echo time (TE) would seem obvious to achieve high T2 weighting, considerably shorter TE has been used in some studies on STIR [27]. Apart from the fact that on some MR systems, the signal-to-noise ratios achievable with long TE are not sufficient to generate diagnostic images when the standard configurations of black-blood pulse sequences provided by the manufacturer are used, the choice of shorter TE might be justified as the contrast between typical remote and edematous myocardium should not change significantly for TE beyond 50 ms (Figure 5).

One concern in relation to STIR imaging that is frequently raised is the fact that slow-flowing blood in the ventricular cavity can give bright signal at the endocardial border. This can be mistaken for myocardial hyperintensity. In our study, we used cine images at the same spatial and temporal location as a reference for endocardial contour detection. This method effectively helps avoid confusion with intracavity signals.

A number of other methods of T2 weighted imaging have recently been described including bright [28,29] and dark blood [30] methods. These have recently been compared and showed significant variation in the size of the area of edema [31]. STIR imaging seems to provide a reasonable balance between sensitivity and specificity when all available clinical and angiographic data are used. In addition, STIR has the advantage of being the most widely available sequence. It has also available for more than 10 years, which results in better knowledge of its use and potential artifacts.

While our study shows the high value of STIR for the qualitative ("yes/no") detection of focal myocardial edema, this technique is limited as any other conventional MR technique in the way that it does not allow for absolute quantification of signal intensity. In other words, the identification of myocardial edema with STIR is based on its perceptible contrast to non-edematous myocardium rather than on absolute thresholds. Therefore, applications involving diffuse processes or serial follow-up (e.g. for the assessment of inflammatory activity) should benefit from the use of alternative MR strategies enabling absolute signal quantification such as T1 mapping [20] or T2 mapping [32,33].

## Conclusions

CMR using STIR enables the detection of infarct-related myocardial edema with high spatial resolution and high diagnostic accuracy. STIR imaging possesses unique properties beyond T2-weighting and fat suppression, and should be regarded as an optimized edema-weighted technique rather than a purely T2-weighted method. CMR centers and individuals interested in the use of STIR should compare their results to those of other centers in order to optimize their protocols, and should not settle for sub-optimal image quality.

## Abbreviations

STIR: Short inversion time inversion recovery; CMR: Cardiovascular magnetic resonance; LGE: Late gadolinium enhancement; MI: Myocardial infarction; MR: Magnetic resonance; Gd: Gadolinium; LV: Left ventricle; SD: Standard deviations; CK: Creatine kinase; MSI: Myocardial salvage index; TSE: Turbo spin echo; TI: Inversion time.

## Competing interests

The authors declare that they have no competing interests.

## Authors' contributions

DOH performed all MRI data analysis and drafted the manuscript. JPR provided advice on the STIR signal behavior. TK and FB helped to design the study. MS and SP supervised patient recruitment and MRI data acquisition. DM recruited the patients, performed the MRI data acquisition, and drafted the manuscript. All authors read and approved the final manuscript.
